# Shortened Lung Clearance Index is a repeatable and sensitive test in children and adults with cystic fibrosis

**DOI:** 10.1136/bmjresp-2014-000031

**Published:** 2014-07-21

**Authors:** David Hannon, Judy M Bradley, Ian Bradbury, Nicholas Bell, J Stuart Elborn, Katherine O'Neill

**Affiliations:** 1 Centre for Infection and Immunity, School of Medicine, Dentistry and Biomedical Sciences, Queen's University Belfast, Belfast, UK; 2 Centre for Health and Rehabilitation Technologies (CHART), University of Ulster, Belfast, UK; 3 Respiratory Medicine Department, Bristol Adult Cystic Fibrosis Centre, Bristol Royal Infirmary, University Hospitals Bristol, Bristol, UK

**Keywords:** Cystic Fibrosis, Equipment Evaluations

## Abstract

**Background:**

Lung clearance index (LCI) derived from sulfur hexafluoride (SF_6_) multiple breath washout (MBW) is a sensitive measure of lung disease in people with cystic fibrosis (CF). However, it can be time-consuming, limiting its use clinically.

**Aim:**

To compare the repeatability, sensitivity and test duration of LCI derived from washout to 1/30th (LCI_1/30_), 1/20th (LCI_1/20_) and 1/10th (LCI_1/10_) to ‘standard’ LCI derived from washout to 1/40th initial concentration (LCI_1/40_).

**Methods:**

Triplicate MBW test results from 30 clinically stable people with CF and 30 healthy controls were analysed retrospectively. MBW tests were performed using 0.2% SF_6_ and a modified Innocor device. All LCI end points were calculated using SimpleWashout software. Repeatability was assessed using coefficient of variation (CV%). The proportion of people with CF with and without abnormal LCI and forced expiratory volume in 1 s (FEV_1_) % predicted was compared. Receiver operating characteristic (ROC) curve statistics were calculated. Test duration of all LCI end points was compared using paired t tests.

**Results:**

In people with CF, LCI_1/40_ CV% (p=0.16), LCI_1/30_ CV%_,_ (p=0.53), LCI_1/20_ CV% (p=0.14) and LCI_1/10_ CV% (p=0.25) was not significantly different to controls. The sensitivity of LCI_1/40_, LCI_1/30_ and LCI_1/20_ to the presence of CF was equal (67%). The sensitivity of LCI_1/10_ and FEV_1_% predicted was lower (53% and 47% respectively). Area under the ROC curve (95% CI) for LCI_1/40_, LCI_1/30_, LCI_1/20_, LCI_1/10_ and FEV_1_% predicted was 0.89 (0.80 to 0.97), 0.87 (0.77 to 0.96), 0.87 (0.78 to 0.96), 0.83 (0.72 to 0.94) and 0.73 (0.60 to 0.86), respectively. Test duration of LCI_1/30_, LCI_1/20_ and LCI_1/10_ was significantly shorter compared with the test duration of LCI_1/40_ in people with CF (p<0.0001) equating to a 5%, 9% and 15% time saving, respectively.

**Conclusions:**

In this study, LCI_1/20_ was a repeatable and sensitive measure with equal diagnostic performance to LCI_1/40_. LCI_1/20_ was shorter, potentially offering a more feasible research and clinical measure.

**Key messages**

- Lung Clearance Index (LCI) can be time-consuming, limiting its use clinically.
- Investigation of the flexibility of current multiple breath washout test end points is an important area for future research.
- LCI_1/20_ is a repeatable and sensitive test that is shorter than LCI_1/40_, potentially offering a more feasible research and clinical measure.

## Introduction

Lung Clearance Index (LCI) derived from multiple breath washout (MBW) is a sensitive measure of ventilation inhomogeneity^1^ ^2^ and a robust surrogate outcome measure of the severity of lung disease in cystic fibrosis (CF)^3^ which has begun to be incorporated into clinical trials.^4^ ^5^ It also shows promise as a sensitive outcome measure in idiopathic bronchiectasis^6^ and asthma.^7^ A drawback of the test is that it can be time-consuming, especially in patients with advanced disease, limiting its feasibility within the clinical environment. By convention a MBW test involves performing a minimum of three inert tracer gas washout runs, ending the washout when end-tidal tracer gas concentration falls below 1/40th of the initial concentration.^8^ The end point of 1/40th is based on historic studies and has not been systematically validated.^8^ ^9^ The European Respiratory Society/American Thoracic Society (ERS/ATS) consensus statement highlights investigation of the flexibility of current MBW test end points as an important area for future research, which could potentially improve the utility of this test.^8^ Assessment of the clinimetric properties of shortened LCI in CF using nitrogen (N_2_) MBW testing (100% as the inert gas), have reported good diagnostic performance in children with mild disease, offering a measure of ventilation inhomogeneity which may be more practical in the clinical setting.^10^ However, there are no studies to assess the performance of shortened LCI using sulfur hexafluoride (SF_6_) MBW (another common MBW method), or studies of shortened LCI in adult patients with more moderate to advanced disease. Differences in gas diffusion and molecular mass of the inert gases used mean that results of the two types of test are not comparable.^11^ Study of the sensitivity of shortened MBW tests using SF_6_ could be useful in improving the clinical utility of these tests.

In this study we aimed to assess and compare the repeatability, sensitivity, specificity and test duration of LCI derived from washout to 1/30th (LCI_1/30_), 1/20th (LCI_1/20_) and 1/10th of the initial concentration (LCI_1/10_) to ‘standard’ LCI derived from washout to 1/40th initial concentration (LCI_1/40_), using 0.2% SF_6_ as the tracer gas, in school age—adolescent children and adults with CF and healthy controls.

## Methods

### Subject recruitment

Cross-sectional data from 30 people with CF (n=15 aged 6–17 years old; n=15 aged ≥18 years old) and 30 healthy control participants (n=15 aged 6–17 years old; n=15 aged ≥18 years old) with three valid and repeatable MBW tests were analysed. Thirty anonymised CF and 30 healthy control data sets, as consecutively listed in a database of results collected in a large prospective project investigating the clinimetric and clinical relevance of LCI in CF were used. People with CF were recruited at a routine outpatient visit to the Northern Ireland paediatric and adult CF centres at Belfast Health and Social Care Trust (BHSCT), when clinically stable (no pulmonary exacerbation requiring intravenous antibiotics in the previous 4 weeks), between October 2010 and June 2013. Control participants were recruited by means of email circulation among people employed in Queen's University Belfast (QUB) and BHSCT between September 2011 and August 2012. All adult participants provided written informed consent. All child participants provided child or young person assent and parental consent.

### MBW testing

The MBW test to measure LCI was carried out using a modified Innocor device and 0.2% SF_6_ using the open-circuit technique in accordance with the standard operating procedure developed by the UK CF Gene Therapy Consortium (UKCFGTC; see online supplementary file 1) as described and validated by Horsley *et al*^2^ and used in a recent CF clinical trial and observational study.^4^ ^12^ Participants breathed through a mouthpiece at tidal volumes, while in a seated position and wearing a nose clip. Participants breathed 0.2% SF_6_ in air via a flowpast circuit until washin was complete, at which point the flowpast was disconnected and the participant breathed room air until the end tidal expired SF_6_ concentration fell below 1/40th of the initial concentration before disconnection. Three washouts were performed for each participant. Analysis of MBW data was performed using the SimpleWashout programme developed by Dr Nicholas Bell (UKCFGTC) and used with his permission (see online supplementary file 1). For each washout, four values for functional residual capacity (FRC) and LCI were calculated:

- FRC_1/40_ and LCI_1/40_ were derived from washout data from flowpast disconnection until the first breath with end tidal SF_6_ concentration below 1/40th (≤0.005%) of the starting SF_6_ concentration (0.2%).
- FRC_1/30_ and LCI_1/30_ were derived from washout data from flowpast disconnection until the first breath with end tidal SF_6_ concentration below 1/30th (≤0.007%) of the starting SF_6_ concentration (0.2%).
- FRC_1/20_ and LCI_1/20_ were derived from washout data from flowpast disconnection until the first breath with end tidal SF_6_ concentration below 1/20th (≤0.01%) of the starting SF_6_ concentration (0.2%).
- FRC_1/10_ and LCI_1/10_ were derived from washout data from flowpast disconnection until the first breath with end tidal SF_6_ concentration below 1/10th (≤0.02%) of the starting SF_6_ concentration (0.2%).

Mean LCI and FRC values and test duration (minutes) for each end point were calculated from each of the three washouts in each testing session.

### Spirometry

Spirometry was measured according to American Thoracic Society/European Respiratory Society ATS/ERS guidelines^13^ using a Microlab (ML3500 MK8) spirometer (CareFusion, Kent, UK). Predicted values were calculated from reference ranges for all ages.^14^

### Statistical analysis

Data were analysed using PASW Statistics (V.18, IBM software, USA) and Prism (V.5.01 GraphPad Software Inc.) packages. CF and control participant characteristics were summarised using descriptive statistics.

Intravisit repeatability of LCI_1/40_, LCI_1/30_ LCI_1/20_ and LCI_1/10_ was assessed using the coefficient of variation (CV%) of all three tests and Bland-Altman plots^15^ comparing tests one and three, for people with CF and healthy controls. Results between people with CF and healthy controls were compared using an independent samples t test. Mean LCI and FRC values for each end point from people with CF or healthy controls were compared using paired samples t tests. The relationship between mean LCI_1/40_ and mean LCI_1/30,_ LCI_1/20_ and LCI_1/10_ was assessed using scatter plots and the Spearman's rank correlation coefficient. This analysis was also used to assess the relationship between FEV_1_% predicted and all LCI end points. Sensitivity of all LCI end points compared with FEV_1_% predicted was assessed using scatter plots and limits of normal of respective tests calculated from healthy controls (mean +1.96 SD). Sensitivity and specificity were further analysed using receiver operating characteristic (ROC) curves, and by comparing area under the ROC curve (AUC_ROC_) and 95% CI for LCI_1/40_, LCI_1/30_, LCI_1/20_, LCI_1/10_ and FEV_1_% predicted. Mean test duration (minutes) of LCI_1/40_, LCI_1/30_, LCI_1/20_ and LCI_1/10_ was compared using a paired samples t test. As multiple comparisons were being made, a Bonferroni adjustment was incorporated. A p value of <0.01 was considered statistically significant.

## Results

LCI_1/40_, LCI_1/30_, LCI_1/20,_ LCI_1/10_ and FEV_1_% predicted were significantly different between the CF and control group. However, there was no difference between the CF and control group in age, sex, LCI_1/40_ CV%, LCI_1/30_ CV%, LCI_1/20_ CV%, LCI_1/10_ CV% or test duration of any LCI end point (table 1).

**Table 1:** CF and healthy control participant characteristics

	People with CF	Healthy controls	p Value (CF vs controls)
N	30	30	–
M/F	14/16	16/14	0.61
Age (years)	20.7 (11.1) (6–51)	20.8 (10.7) (7–44)	0.93
FEV_1_ (% predicted)	79.3 (17.9) (46.0–116.0)	92.9 (11.3) (68.0–116.0)	0.009
FRC_1/40_ (L)	1.95 (0.76)	2.23 (1.0)	0.22
LCI_1/40_ (number of turnovers)	9.0 (2.4)	6.4 (0.5)	<0.0001
LCI_1/40_ CV%	5.2 (2.8)	4.3 (2.0)	0.16
LCI_1/40_ triplicate test duration (min)	21.8 (9.7) (8.0–57.1)	19.4 (6.5) (10.1–31.1)	0.26
FRC_1/30_ (L)	1.93 (0.76)	2.24 (1.02)	0.18
LCI_1/30_ (number of turnovers)	7.7 (1.6)	5.9 (0.4)	<0.0001
LCI_1/30_ CV%	4.6 (3.2)	4.1 (2.8)	0.53
LCI_1/30_ triplicate test duration (min)	20.9 (9.1) (7.3–53.2)	18.9 (6.2) (9.9–29.9)	0.34
FRC_1/20_ (L)	1.90 (0.75)	2.24 (1.0)	0.15
LCI_1/20_ (number of turnovers)	6.6 (1.2)	5.2 (0.4)	<0.0001
LCI_1/20_ CV%	5.7 (3.4)	4.6 (2.3)	0.14
LCI_1/20_ triplicate test duration (min)	20.0 (8.9) (6.7–51.7)	18.3 5.9 (9.6–28.3)	0.38
FRC_1/10_ (L)	1.84 (0.73)	2.22 (1.00)	0.09
LCI_1/10_ (number of turnovers)	4.7 (0.6)	4.0 (0.3)	<0.0001
LCI_1/10_ CV%	5.4 (3.8)	4.3 (2.8)	0.25
LCI_1/10_ triplicate test duration (min)	18.6 (8.1) (5.7–46.9)	17.1 (5.5) (8.8–26.6)	0.41

All values summarised as mean (SD)±(range).

CF, cystic fibrosis; CV, coefficient of variation, F, female; FEV_1,_ forced expiratory volume in 1 s; FRC, functional residual capacity; LCI, Lung Clearance Index; M, male.

There was no difference between the CF and control group in any of the FRC values (FRC_1/40_, FRC_1/30_, FRC_1/20_ or FRC_1/10_). Within the CF group, as expected, FRC was incrementally lower with each earlier end point. Although FRC_1/40_, and FRC_1/30_ were not significantly different (1.95 vs 1.93, p=0.07) FRC_1/40_ and FRC_1/20_ (1.95 vs 1.90, p=0.002) and FRC_1/40_ and FRC_1/10_ (1.95 vs 1.84) were significantly different.

### Intravisit repeatability

**Figure 1:**
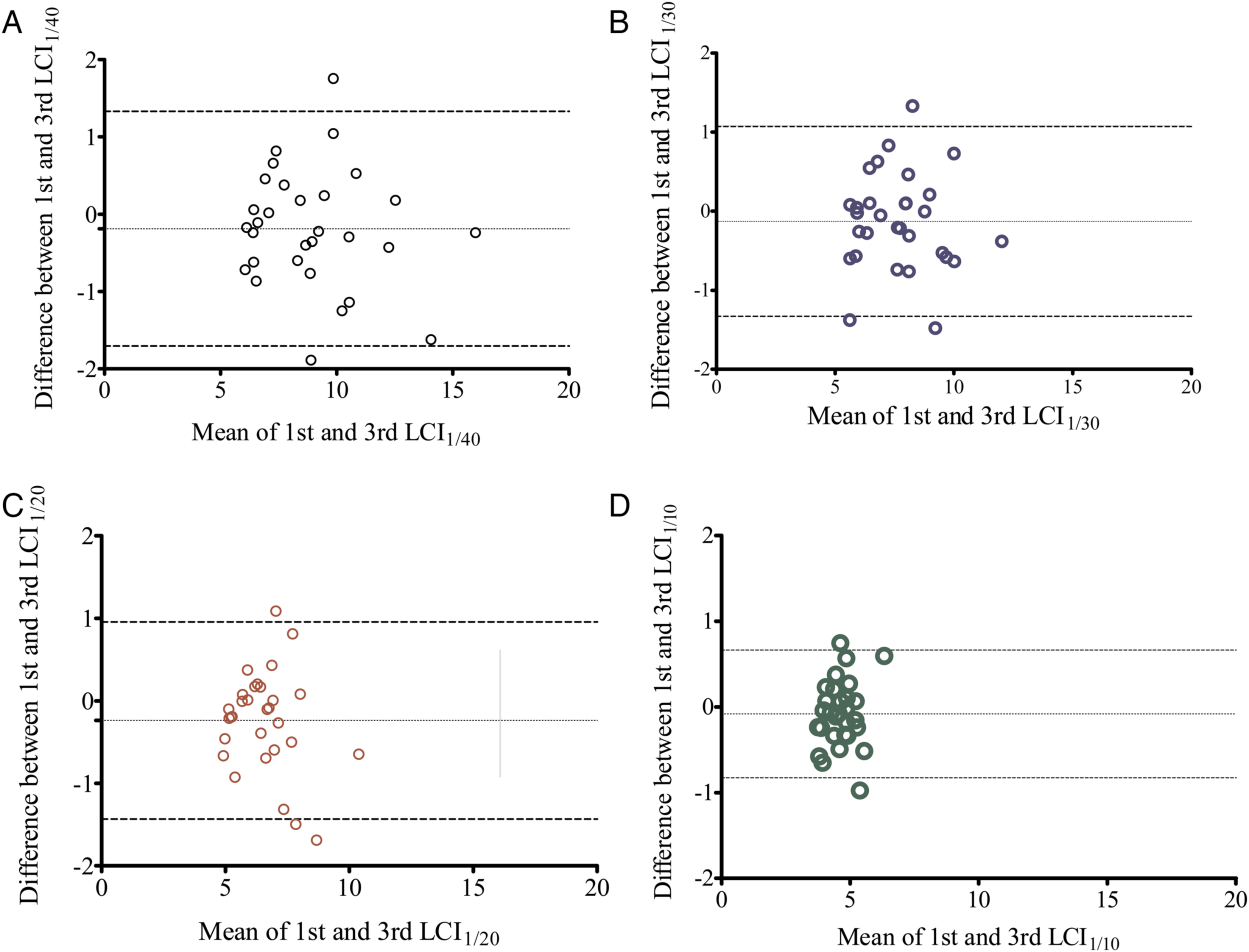
(A) Lung Clearance Index (LCI)1/40; (B) LCI1/30; (C) LCI1/20; (D) LCI1/10 first and third test in people with cystic fibrosis (dotted horizontal lines represent the bias and 95% limits of agreement).

LCI_1/40_ CV%, LCI_1/30_ CV%, LCI_1/20_ CV% and LCI_1/10_ CV% in people with CF were not significantly different to values in healthy controls (table 1). There was also no significant difference between LCI_1/40_ CV% and the CV% of any other LCI end point in people with CF. A Bland-Altman plot^15^ of the mean versus the difference between tests one and three of LCI_1/40_, LCI_1/30,_ LCI_1/20_ and LCI_1/10_ for people with CF showed no evidence of greater variability in participants with more advanced disease (ie, a higher LCI reading; figure 1A–D).

For LCI_1/40_, the 95% limits of agreement between the two measurements were −1.70 to 1.33 lung turnovers, compared with −1.33 to 1.07 (LCI_1/30_), −1.43 to 0.96 (LCI_1/20_) and −0.83 to 0.66 (LCI_1/10_) lung turnovers. Therefore the intravisit repeatability of the LCI_1/40_, LCI_1/30_, LCI_1/20_ and LCI_1/10_ measurements was 1.5, 1.2, 1.2 and 0.7 lung turnovers respectively.

### Relationship between shortened LCI and ‘standard’ LCI_1/40_

**Figure 2:**
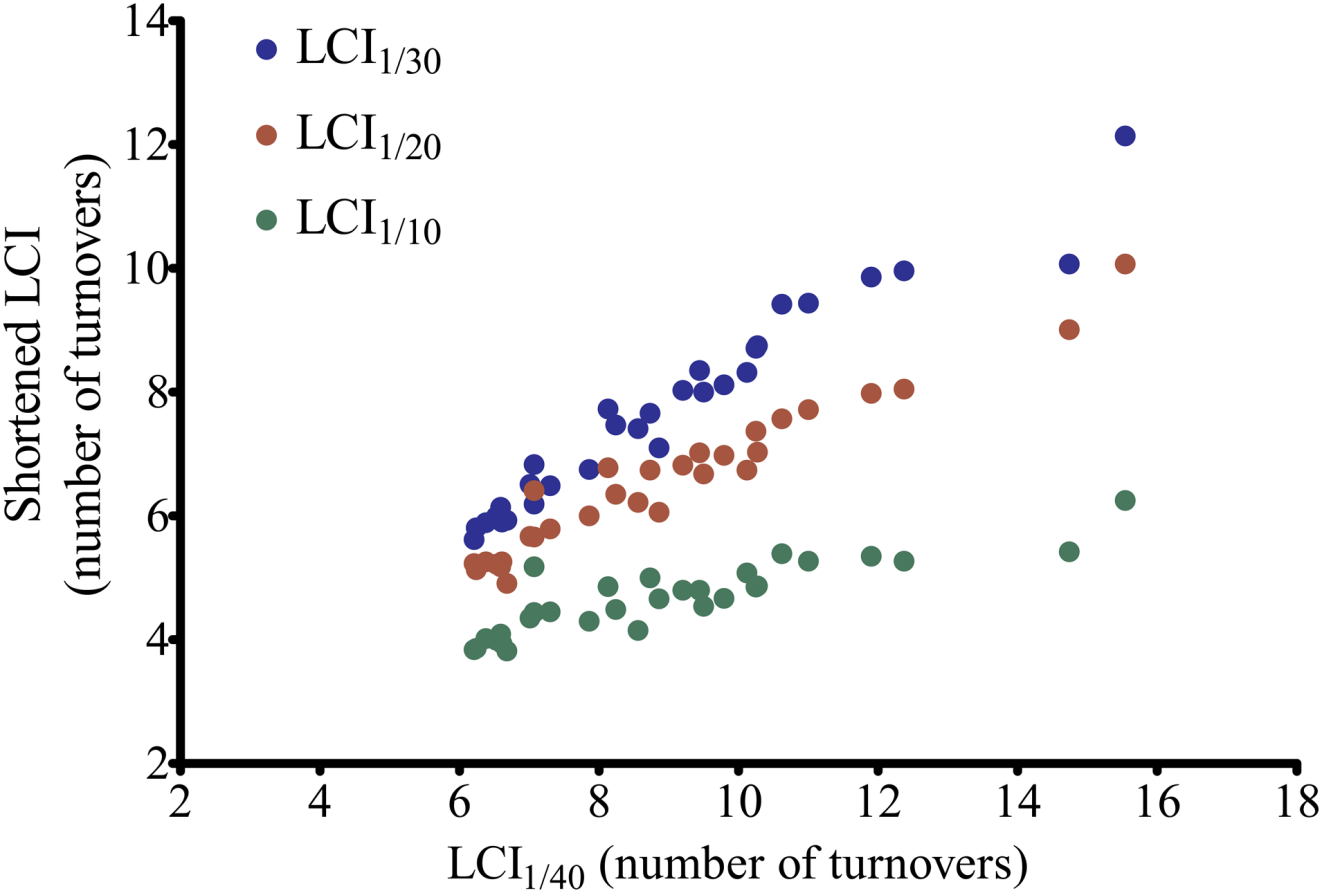
Shortened Lung Clearance Index (LCI) versus standard LCI1/40 in people with cystic fibrosis.

In people with CF, LCI_1/30_ (r=0.98, p<0.0001), LCI_1/20_ (r=0.95, p<0.0001) and LCI_1/10_ (r=0.88, p<0.0001) correlated significantly with LCI_1/40_ (figure 2).

### Sensitivity and specificity

The upper limit of normal for LCI_1/40_, LCI_1/30_, LCI_1/20_ and LCI_1/10_ was 7.3, 6.7, 5.9 and 4.6 lung turnovers, respectively, (control mean +1.96 SD). The lower limit of normal of 80% for FEV_1_% predicted was used, as this is the level that is historically used in clinical practice.

**Figure 3:**
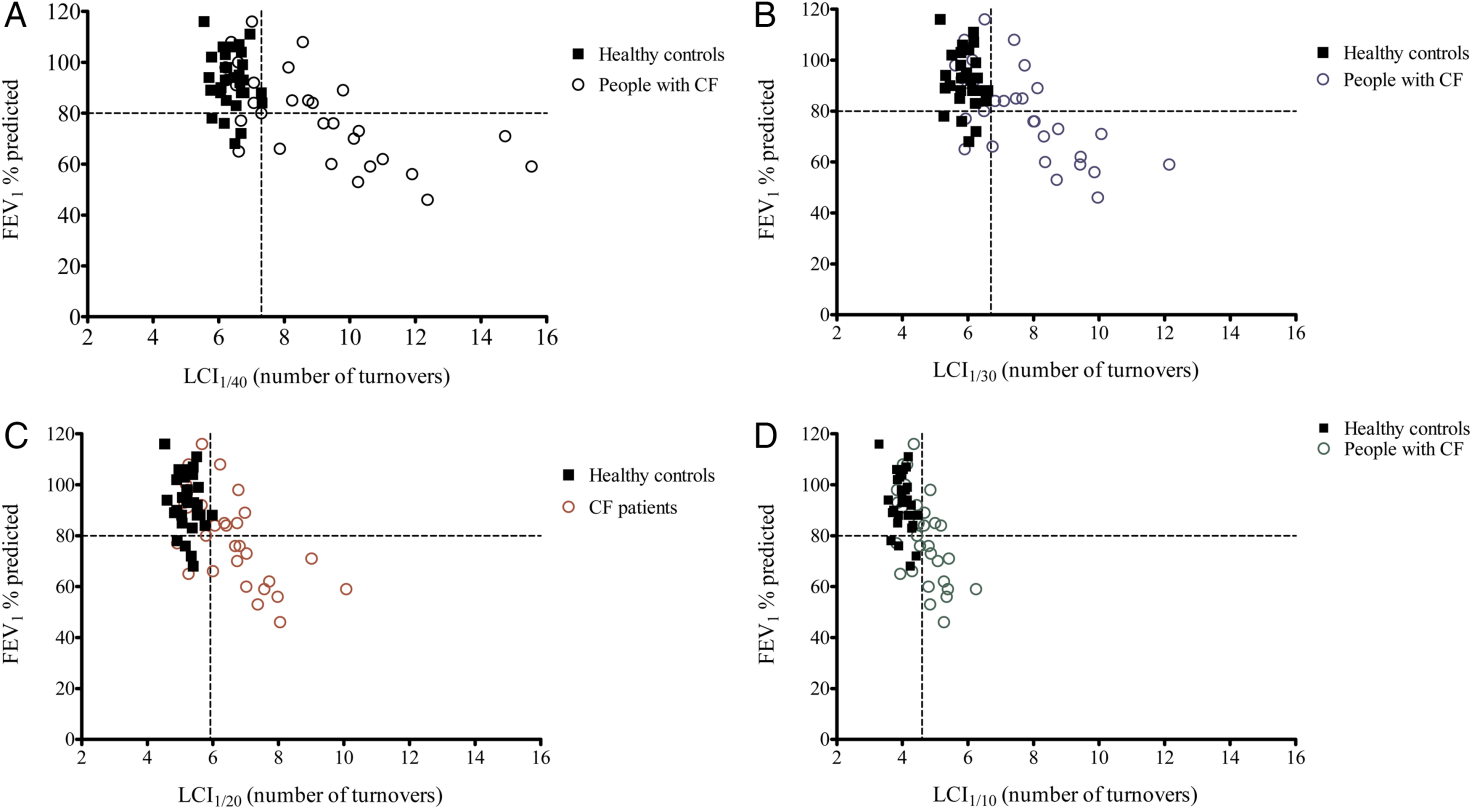
Forced expiratory volume in 1 s (FEV1)% predicted versus (A) LCI1/40; (B) LCI1/30; (C) LCI1/20 (D) LCI1/10 (dotted horizontal lines represent the limits of normal for FEV1% predicted (80% predicted) and LCI (LCI1/40:7.3; LCI1/30:6.7; LCI1/20:5.9; LCI1/10:4.6)).

The sensitivity of LCI_1/40_, LCI_1/30_, LCI_1/20_ to differentiate between people with CF and healthy controls was identical (67%). The sensitivity of LCI_1/10_ and FEV_1_% predicted was lower (53% and 47%, respectively). In people with CF, LCI_1/40_ (r=−0.73, p<0.0001), LCI_1/30_ (r=−0.70, p<0.0001), LCI_1/20_ (r=−0.69, p<0.0001) and LCI_1/10_ (r=−0.62, p=0.0003) correlated significantly with FEV_1_% predicted (figures 3A–D). Using LCI_1/40_, 6/30 (20%) people with CF had an abnormal LCI in the presence of a normal FEV_1_% predicted (figure 3A). Similarly, using LCI_1/30_ or LCI_1/20_, 7/30 (23%) people with CF had an abnormal LCI in the presence of a normal FEV_1_% predicted (figure 3B–C). LCI_1/10_ was less sensitive, detecting 5/30 (17%) with an abnormal LCI in the presence of a normal FEV_1_% predicted (figure 3D).

**Figure 4:**
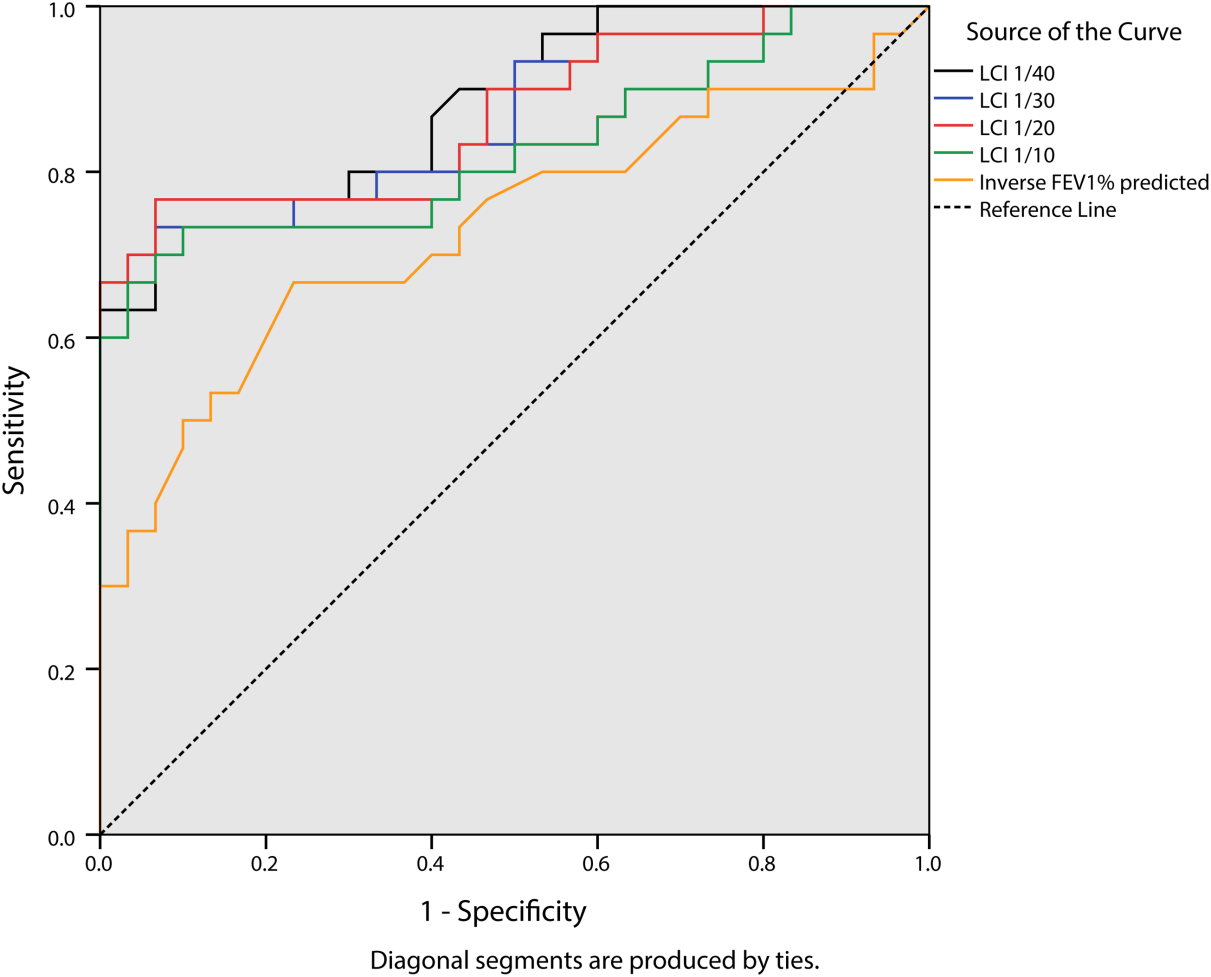
Receiver operating characteristic ROC curve of Lung Clearance Index (LCI)1/40, LCI1/30, LCI1/20, LCI1/10 and inverse forced expiratory volume in 1 s % predicted: sensitivity and specificity to the presence of cystic fibrosis CF.

ROC curve analysis (figure 4) showed that while all LCI values and FEV_1_% predicted had statistically significant levels of sensitivity and specificity in determining people with CF vs control participants, LCI_1/40,_ LCI_1/30_ and LCI_1/20_ had comparable and higher sensitivity and specificity compared with LCI_1/10_ and FEV_1_% predicted (table 2).

**Table 2:** AUC_ROC_ and 95% CI for LCI_1/40_, LCI_1/30_, LCI_1/20_ LCI_1/10_ and inverse FEV_1_% predicted (1.0 indicating best performance, 0.5 indicating poor performance)

	AUC_ROC_	95% CI	p Value
LCI_1/40_	0.89	0.80 to 0.97	<0.0001
LCI_1/30_	0.87	0.77 to 0.96	<0.0001
LCI_1/20_	0.87	0.78 to 0.96	<0.0001
LCI_1/10_	0.83	0.72 to 0.94	<0.0001
FEV_1_% predicted (inverse)	0.73	0.60 to 0.86	0.002

AUC, area under the curve; FEV_1,_ forced expiratory volume in 1 s; LCI, Lung Clearance Index, ROC, receiver operating characteristic

### Test duration

Test duration of LCI_1/30,_ LCI_1/20_ and LCI_1/10_ was significantly shorter compared with washout duration of LCI_1/40_ in people with CF (p<0.0001) and in healthy controls (p<0.0001; table 1). In people with CF, the mean (95% CI) time saving per triplicate MBW test was 1 (0.8 to 1.3) minutes or 5% with LCI_1/30_, 1.9 (1.4 to 2.3) minutes or 9% with LCI_1/30_ and 3.3 (2.6 to 4.2) minutes or 15% with LCI_1/10_.

## Discussion

This study is the first to show that SF_6_ MBW tests can be reliably shortened. Results show that in children and adults with CF, LCI shortened to 1/30th or 1/20th (LCI_1/30_ or LCI_1/20_) of the initial concentration have comparable intravisit repeatability and sensitivity to ‘standard’ LCI at 1/40th of the starting concentration (LCI_1/40_) providing additional information to FEV_1_% predicted and offering a time saving. Although repeatable, LCI shortened to 1/10th of the starting concentration (LCI_1/10_) was less sensitive to lung disease, compared with the other LCI end points. It was, however, still more sensitive than FEV_1_% predicted.

The ‘standard’ end point of 1/40th is based on historic studies using nitrogen washout (2.5%) and has not been systematically validated for MBW tests using SF_6_.^8^ ^9^ This study aimed to assess the performance of earlier arbitrary end points compared with the ‘standard’ end point in the SF_6_ washout, in an attempt to improve the clinical utility of the MBW test by reducing test duration. Like LCI_1/40_, LCI_1/30_ and LCI_1/20_ target the flatter tail of the washout curve, making it unsurprising that similar information can be obtained (see online supplementary file 2). In contrast, when using LCI_1/10,_ the end point occurs before the washout curve flattens. This supports the theory that most information is contained in the tail of the washout curve.^16^ Therefore a cut-off before this point may provide less information about lung disease severity, as highlighted by the lower sensitivity of LCI_1/10_ in this study_._ Yammine *et al*^10^ assessed the repeatability and sensitivity of shortened N_2_ MBW to measure LCI, at a number of earlier end points including 1/20th of the starting concentration and as early as 1/5th of the starting concentration, in 68 children with CF (n=44 with mild disease). In agreement with results from our study, they reported good performance of shortened LCI at 1/20th of the starting concentration compared with ‘standard’ LCI. Furthermore, as with the results from our study, Yammine *et al*^10^ found that while the earlier LCI end points had good intravisit repeatability, they were less sensitive and specific to the presence of CF. The authors concluded that shortened N_2_ MBW to 1/20th of the starting concentration could offer a more feasible measure for use in clinical practice. In this study we extended these observations to investigate and confirm the utility of shortened ventilation indices in older patients with more advanced disease. Theoretically, in cases of severe flow asynchrony between the best and the least ventilated lung units, the end-tidal concentrations in subsequent breathing cycles can enhance the contribution of the least ventilated units toward the end of the washout where LCI is measured.^16^ This may lead to increased variation in results taken at an earlier end point, such as 1/20th of the starting concentration. In contrast, LCI from an earlier end point may be more precise, as an end point where the washout curve slope is greater may avoid random breath-by-breath variability that could be observed at a later end point. In this study, LCI_1/30_ and LCI_1/20_ had marginally better sensitivity to the presence of CF (23% vs 20%), compared with LCI_1/40_, even though there was no significant difference in variability (LCI CV%) between ‘standard’ LCI (LCI_1/40_) and LCI at an earlier end point (LCI_1/30_, LCI_1/20_, LCI_1/10_). LCI_1/30_ and LCI_1/20_ also had good diagnostic performance and superior sensitivity compared with FEV_1_% predicted. Importantly, these findings indicate that no further additional information was obtained using LCI_1/40_ compared with the shortened versions, LCI_1/30_ and LCI_1/20_.

Test duration for LCI_1/30_, LCI_1/20_ and LCI_1/10_ was significantly shorter than LCI_1/40_ in people with CF, with an average 5%, 9% and 15% time saving, respectively. As LCI_1/20_ offers a greater time saving than LCI_1/30,_ while maintaining reliability and sensitivity, it provides the most attractive measure and may enhance the feasibility of MBW in the research and clinical setting. The proportion of time saved in this study is smaller than that reported in the study by Yammine *et al*^10^ as MBW tests using an exogenous gas such as SF_6_ require a washin and a washout phase. However, as these results are from a retrospective analysis, the time saving measurement does not take into account the total time saving per testing session. Finishing a test earlier would allow for the second and third test of the triplicate to start sooner, resulting in a larger time saving. Although the washin time is unchanged, any shortening of LCI test duration could be especially useful in younger children where long assessment periods are not feasible, and in patients with advanced disease, where the washin and/or washout periods can be prolonged. The use of MBW equipment using SF_6_ has been successfully used in multicentre studies and remains popular as it has advantages in terms of tracer gas estimation (measured directly rather than by subtraction as with N_2_ MBW) avoiding the potential confounding effects of 100% O_2_ on breathing pattern. This study is the first to show that MBW using SF_6_ can be reliably shortened.

Considering potential limitations of shortened MBW tests, one study highlights that advanced analysis of washout curves to determine the relative contribution of convective and acinar airways to ventilation heterogeneity (phase III analysis) usually requires six lung-volume turnovers.^17^ However, recent work suggests that the same information may be obtainable in three lung-volume turnovers^18^ in which case use of LCI_1/20_ would still enable full phase III analysis. Regardless, phase III indices may have limited utility in CF, as demonstrated by Horsley *et al*.^19^

The retrospective nature of this study is a limitation. However, the study did endeavour to avoid selection bias by use of anonymsied patient data sets as consecutively listed in a database and represents the first exploratory study to report on the clinimetric properties of an earlier end point in SF_6_ MBW. More data from across the disease severity range in CF are required to define normal ranges of shortened LCI.

## Conclusions

LCI_1/20_ is a repeatable and sensitive test with equal diagnostic performance to LCI_1/40_ that is shorter, potentially offering a more feasible research and clinical measure.

## Supplementary Material

Web supplement

Web supplement
