# Supplementary material for: Shortened Lung Clearance Index is a repeatable and sensitive test in children and adults with cystic fibrosis
Source: BMJ Open Respir Res. 2014 Jul 21;1(1):e000031. doi: 10.1136/bmjresp-2014-000031 (PMC4212720; doi:10.1136/bmjresp-2014-000031)
Supplement: Web supplement [file bmjresp-2014-000031-s1.pdf]

Online supplement 1

The Multiple Breath Washout (MBW) test to measure Lung Clearance Index (LCI) values was carried out using the modified Innocor^{TM} device and 0.2% sulfur hexafluoride (SF_6) using the open-circuit technique in accordance with the standard operating procedure developed by the UK CF Gene Therapy Consortium (UKCFGTC).

Equipment setup

The Innocor^{TM} equipment set-up to measure functional residual capacity (FRC) and LCI is described in detail in the original validation paper (Horsley et al., 2008).

The adapted patient interface and the flowpast circuit consisted of a mesh type flowmeter (Model 4700A non-heated; flow range 0-160L/min, Hans Rudolph, Kansas, USA), a Intersurgical Clear Guard II bacterial filter (Surgical systems Ireland, Craigavon, N. Ireland ref 1844) and a silicone mouthpiece (Hans Rudolph; Ferraris, UK; size small) fitted over the filter end. Total pre-capillary deadspace (between the gas sampling needle and the patient's mouth) of the adult patient interface was calculated to be 68.2mls from derived component volumes (flowmeter 14.2mls; filter 54mls). Total pre-capillary deadspace of the children's patient interface was calculated to be 48.5mls from derived component volumes (flowmeter 18.5mls; filter 30mls).

A hole was drilled through the small flange side port tube of the flowmeter to house the gas sampling needle (shortened and blunted 21 gauge needle) which was then attached to the gas sampling line. This was positioned distal from the mouthpiece, to avoid the possibility of the gas sampling needle interfering with the functioning of the flowmeter and to reduce re-inspired SF_6. The gas sampling line was made from Nafion tubing, allowing dry gas concentrations to be calculated. Post-capillary deadspace (space between the gas sampling needle and end of flowmeter port) was calculated at 5mls equating to less than 1% of the typical minimum tidal volume.

The flowpast circuit consisted of a T-piece connector (22mm-22F-22mm; Intersurgical, Surgical systems Ireland, Craigavon, N. Ireland) which attached to the end of the flowmeter port. The T-piece was attached to disposable plastic anaesthetics tubing (Hudson RCI. Respiratory and anesthesia products Corr-A-flex II 22mm tubing Ref1680) with a reservoir bag (Intersurgical 3 litre 22F neck) in the upstream arm. The reservoir bag was connected to the cylinder of compressed 0.2% SF_6 in air. A fan was in place to disperse any SF_6 expired during the washout phase in order to prevent re-inspiration and subsequent effect on the length of washout and calculation of LCI.

Before each test, the flowmeter was linearised and calibrated. The flow gas delay (FGD) was calculated using inbuilt Innocor^{TM} software (version 6.15). During the test, the Innocor^{TM} software provides visual display of gas concentration, flow and tidal volume allowing identification of the end of wash-in and washout phases and any immediate technical issues.

The modified Innocor^{TM} device meets a number of key recommendations as outlined in the ERS/ATS IGW measurement consensus statement (Robinson et al., 2013). These include:

- Accurate flow measurement within 1% across a range of flows.
- No volume drift in stable gas concentrations.
- Gas analyser accuracy with excellent signal quality.
- Data sampling frequency of 100Hz.

- Accurate synchronisation of gas and flow signals.

(Horsley et al., 2008b).

Performing the test

The test was performed either before or at least 30 minutes after performing spirometry to avoid any effects from a recent forced expiratory manoeuvre. The patient was seated, wearing a noseclip, and asked to breathe normally. Quiet tidal breathing was encouraged by distraction by watching an age-appropriate DVD. The investigator monitored the Innocor^{TM} online display of flow, breath volume, minute volume and gas concentration to ensure a steady breathing pattern. During the wash-in phase the flowmeter was connected to the flowpast T-piece and the subject breathed a mixture of 0.2% SF_6 in dry air, continuing until the Innocor^{TM} online display indicated that the difference between end-inspiration and end-expiration SF_6 was 0.003% or less for three successive breaths. The flowpast T-piece was disconnected from the flowmeter early in expiration during a subsequent breath and the flowpast shut off, the washout phase beginning with the next inspiration. Washout continued until the maximum expired concentration of SF_6 on the Innocor online display was 0.003% or less for three successive breaths. At least three MBW tests were performed at each visit.

Analysis of MBW data

Innocor^{TM} generates raw flow and gas concentration data. These files were uploaded into the SimpleWashout (SW) programme written in the data analysis package Igor Pro, developed by Dr Nicholas Bell (UKCFGTC) and used with his permission. The SW programme was used to calculate FRC and subsequently LCI.

The programme displays the full wash-in and washout, with flow (L/s) and SF_6 concentration (%) signals over time (s), re-aligned according to FGD derived from Innocor software. Breath volume is derived from integration of the flow, and total SF_6 volume is derived from the integration of flow and gas concentration. The programme displays breath by breath data allowing the calculation of FRC and LCI at 1/40th, 1/30th, 1/20th and 1/10th end points.

Each test was individually assessed for validity using quality criteria as facilitated by the SW programme. Quality criteria were as follows:

- Good quality disconnection: Ensuring disconnection during expiration with no leaks, re-breathing or irregular descent of SF_6.
- Complete wash-in: Complete wash-in was defined as a concentration of SF_6 of ≤0.003% for three consecutive breaths. The SW programme calculates a "Cwashin" value (from SF_6 concentration at the end of the last inspiration before disconnection minus the concentration of SF_6 at the end of the last expiration. A "Cwashin" value of ≤ 0.004% was considered ideal; however, values of < 0.008% were considered for inclusion in the calculation of the mean values.

Calculation of FRC_{mbw} & LCI:

- For calculation of $LCI_{1/40}$: the last breath of the washout is defined as the first with an end tidal SF_6 concentration of ≤1/40th of the starting concentration which is followed by two subsequent breaths meeting the same criterion.
- For calculation of $LCI_{1/30}$: the last breath of the washout is defined as the first with an end tidal SF_6 concentration of ≤1/30th of the starting concentration which is followed by two subsequent breaths meeting the same criterion.
- For calculation of $LCI_{1/20}$: the last breath of the washout is defined as the first with an end tidal SF_6 concentration of ≤1/20th of the starting concentration which is followed by two subsequent breaths meeting the same criterion.

- For calculation of $LCI_{1/10}$: the last breath of the washout is defined as the first with an end tidal SF_6 concentration of ≤1/10th of the starting concentration which is followed by two subsequent breaths meeting the same criterion.
- FRC_{mbw} was calculated by dividing the total expired volume of SF_6 over the course of the washout (with last breath defined as in the previous two paragraphs) by [initial SF_6 concentration before disconnection *minus* end tidal expired SF_6 concentration at the end of the last breath].
- The cumulative expired volume (CEV) is the total expired volume over the course of the washout (with last breath in the washout defined as in the previous two paragraphs).
- **LCI = CEV / FRC_{mbw}**
- All tests were then checked for repeatability. FRC values between tests should be within 10% of each other and LCI values between tests should be within 20% of each other. Finally, all mean FRC and LCI value in this study were calculated from a minimum of three valid and repeatable tests.
