# Supplementary material for: Shortened Lung Clearance Index is a repeatable and sensitive test in children and adults with cystic fibrosis
Source: BMJ Open Respir Res. 2014 Jul 21;1(1):e000031. doi: 10.1136/bmjresp-2014-000031 (PMC4212720; doi:10.1136/bmjresp-2014-000031)

Supplementary FIgure

Example of washout curve to demonstrate that washout to 1/40th, 1/30th and 1/20th target the "long tail" of the washout curve.

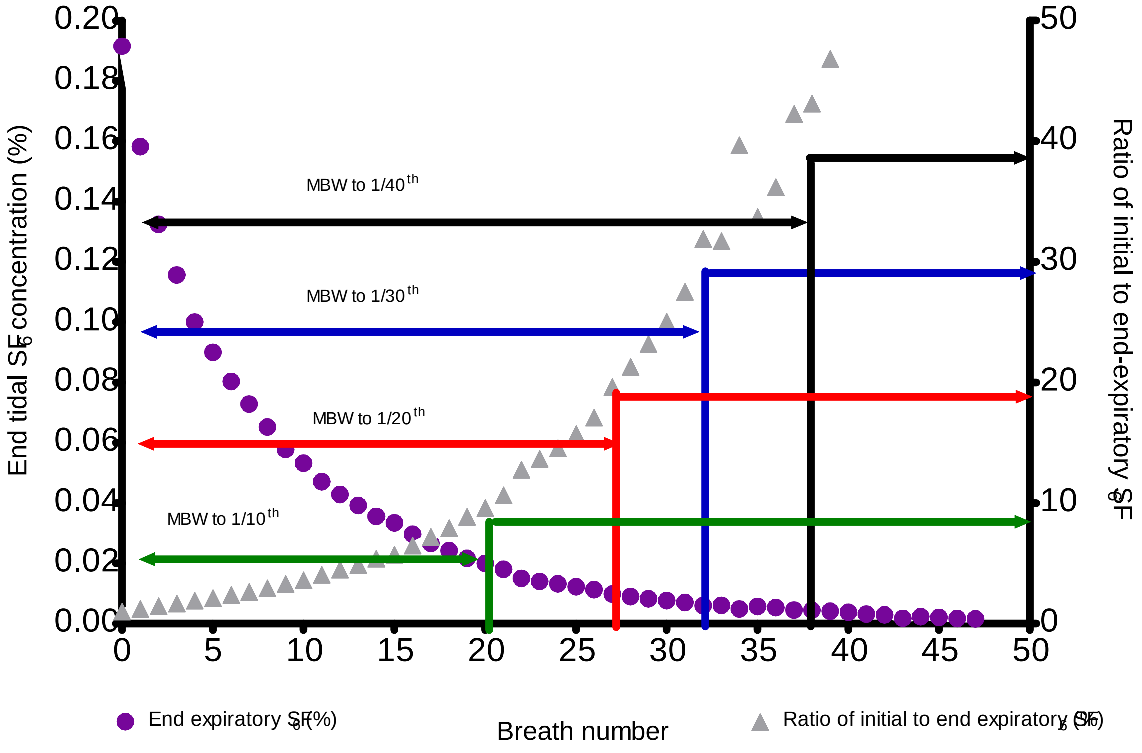

Supplement: Web supplement [file bmjresp-2014-000031-s2.pdf]
